# Standardization of Radon Measurements: II. Accuracy and Proficiency Testing

**DOI:** 10.6028/jres.095.021

**Published:** 1990

**Authors:** J. M. Matuszek

**Affiliations:** Wadsworth Center for Laboratories and Research, New York State Department of Health, Albany, NY 12201

**Keywords:** alpha-track detector, calibration, charcoal detector, electret detector, proficiency test, quality assurance, radon measurement, track-etch detector, uncertainty

## Abstract

The accuracy of *in situ* environmental radon measurement techniques is reviewed and new data for charcoal canister, alpha-track (track-etch) and electret detectors are presented. Deficiencies reported at the 1987 meeting in Wurenlingen. Federal Republic of Germany, for measurements using charcoal detectors are confirmed by the new results. Accuracy and precision of the alpha-track measurements laboratory were better than in 1987. Electret detectors appear to provide a convenient, accurate, and precise system for the measurement of radon concentration. The need for comprehensive, “blind” proficiency-testing programs is discussed.

## 1. Introduction

Since the 1987 meeting of the International Committee for Radionuclide Metrology (ICRM) Low Level Measurements Group in Wurenlingen, Federal Republic of Germany, the New York State Department of Health (NYSDH) has continued to evaluate the performance of *in situ* radon measurement devices available to homeowners and other users: charcoal canisters, alpha-track detectors (ATD) and electret. We have been concerned with the potential accuracies of the available measurement techniques, as well as the effectiveness of on-going proficiency testing programs that aim to provide a link between these measurements and national standards.

## 2. Commercial Radon Measurements in the United States

Most of the routine radon measurements performed in the United States are made by commercial laboratories in an extremely competitive market which has caused price to be a major factor in the selection of a radon-measuring system. Unfortunately, there are many uncertainties which can contribute to the quality of data generated by various radon-measurement systems.

### 2.1 Charcoal Detectors

Charcoal detectors have been found to be unreliable for measurements, because of the very short integrating period [1] as well as the high degree of variability in radon adsorption efficiency caused by displacement of adsorbed radon from the charcoal by water which is also adsorbed during the collection period [1, 2].

Since the Wurenlingen meeting, we have tested four more sets of charcoal detectors using the radon calibration chamber operated by the Environmental Measurements Laboratory (EML) of the U.S. Department of Energy [3], Three of the detector sets were obtained from two commercial laboratories: 7-cm diameter by 2.5-cm thick charcoal canisters from Laboratory A^1^ and two sets of 10-cm diameter by 3-cm thick charcoal canisters, differing by the type of charcoal used as adsorbent, from Laboratory B. A set of 10-cm diameter by 3-cm thick canisters prepared in the Wadsworth Center for Laboratories and Research (WCLR), NYSDH, were also tested. All expose a screened, fully open face for adsorption of radon. Sets of three detectors of each type were exposed in the EML radon chamber for 1,3,4,5, and 7 d, respectively—a total of 15 detectors of each type. Exposed detectors were measured gamma-spectrometrically on a calibrated Ge(Li) system. Also, results published by Ronca-Battista [2] for 10-cm diameter by 3-cm thick charcoal detectors were compared to the data we obtained.

Two of the tested detector sets. Laboratory A and WCLR, use Calgon charcoal, as does the U.S. Environmental Protection Agency (EPA) [2]. Radon adsorption onto the WCLR detectors correlates well with that obtained by Ronca-Battista. The Laboratory A detectors, with approximately half the amount of charcoal, adsorb approximately half as much radon as do the WCLR and EPA detectors for each exposure period. The amount of radon adsorbed per gram of charcoal is approximately the same for each of the three sets of detectors, but the Calgon charcoal used by the WCLR adsorbs approximately twice as much water per gram as do the other two.

The two sets of Laboratory B detectors which use charcoal of unspecified origin showed markedly differing behavior from one another for both radon and water adsorption, apparently due to different brands of charcoal used as adsorbent. The charcoal in the newest Laboratory B detectors has more ^137^Cs activity than other charcoals we have tested. Although the ^137^Cs activity could interfere with radon-daughter activity measurements of exposed canisters. Laboratory B uses high-resolution gamma-spectrometric analysis measurements and hence ^137^Cs presumably does not interfere. The new type of charcoal used by Laboratory B resulted in radon and water adsorption similar to that for the WCLR Calgon-loaded detectors. The charcoal used in the canisters available earlier from Laboratory B showed very poor radon adsorption and very high water uptake—performance very much like that exhibited by the bag type of detector described previously [1]. The new Laboratory B detectors, therefore, are superior to the old ones and approximately equivalent to those used by Laboratory A, EPA, and WCLR in the time over which they can be exposed.

These studies on charcoal detectors add to our previous evaluation [1] that the exposure time for charcoal detectors (at most, 2 to 4 d) is too short to provide a representative measurement of radon concentration in a home. Furthermore, it appears that selection of the wrong type or quantity of charcoal or container may compound the problem of short exposure time such that measurements using certain types of charcoal detectors may be in error by much more than tenfold from the actual radon concentration. (Since publication of the Wurenlingen proceedings [1], a representative of a particular company telephoned to state that the company has ceased using the bag-type detectors because they are considered unreliable. Other companies still are providing bag-type detectors for commercial measurements, however.)

### 2.2 Alpha-Track (Track-Etch) Detectors

The State of New York also continues to contract for radon measurements using alpha-track detectors (ATD), also called track-etch detectors. Although the price per measurement is greater for an ATD than for one using a charcoal detector, recognition of the need for confirmatory measurements using a long-term integrating device requires that the State purchase the more expensive ATDs. So far, our tests of ATDs have been limited. As reported previously [1], when Laboratory C bought out one of its competitors, the competitor laboratory’s customers were required to submit exposed ATDs to Laboratory C for reading. The results were unreliable, with values ranging over an order of magnitude from the low value to the high and differing from the reference value by × 1.5 and ÷8. Laboratory C ATDs read by Laboratory C were tested for this study and the results are listed in table 1. Exposures at EML were as previously described [1]. Various combinations of the exposed ATDs were mailed to Laboratory C on four separate days, because of processing deficiencies noted previously [1]. This time, however, the date of mailing did not produce a statistically significant variation in results, although we do not have information as to the actual date that Laboratory C processed each of the ATDs. There appears to be an overall negative bias in the ATD results of approximately 15%. The lowest values were only 65% of the reference value and none exceeded it.

We have not tested whether other, smaller laboratories which use other types of ATDs, can produce equally or more reliable results.

### 2.3 Electret Detectors

Other types of radon-measuring devices are for the most part either non-integrating, too bulky or fragile to mail, or too expensive to compete in cost with charcoal detectors or ATDs. The possible exception is the electret detectors.

An electret detector system, manufactured by a commercial company [4], was made available to us just one month prior to this symposium. The system is based on an electret detector made of a thin Teflon disc to which a charge of approximately 700 to 750 V is applied and which is then placed into a specially designed chamber. A separate reader is used to measure the charge on the disc prior to exposure and again after exposure to determine the loss of charge due to ionizations caused by impinging alpha particles. Charge loss is then equated to radon concentration using previously developed calibration factors and other correction factors. The results of exposing 15 short-term (typically exposed in a home for no more than 30 d) and 15 long-term (usually exposed for a few months to a year) electret detectors are summarized in table 2.

The electret detectors are convenient to use. Within 2 h of beginning to process the electret detectors for the first time in our laboratory, measurements were completed, results calculated, and data tabulated. In this instance we used a reader, calibration equation and calculational program provided by the manufacturer.

One of the long-term detectors exposed for 3 d exhibited an abnormally high rate of discharge. Although it was suggested by the manufacturer that this may have occurred from someone inadvertently touching the disc when it was removed for reading, further charge measurements indicate that the high discharge rate continued during the following month. This potential source of error remains to be resolved, but one poor measurement out of 30 is a relatively low incidence of inaccurate data compared to the other systems studied. Also, from the standpoint of risk evaluation or remediation requirements, the false positive result created by excess loss of charge is not as serious as a false negative measurement. Presumably, a false positive value would be detected by follow-up measurements. Kotrappa et al. [4] provide a detailed discussion of the advantages and disadvantages of electret detectors.

Uncertainties (expressed as the standard deviation for replicate measurements) are small for the short-term electrets (table 2) even to exposures of 300 pCi d/L. There does not appear to be any significant bias in the short-term results. Although many values in table 2 are slightly negative relative to the corresponding reference value, they mostly seem to be within the statistical uncertainties [4] to be expected for this system.

The results for the long-term electret detectors were initially biased low by approximately 10%. When questioned, the manufacturer found from other users as well that the supplied calibration equation produced low results. A new calibration equation, which increased the long-term results by 10%, was sent to users of the system. The corrected results are the ones listed in table 2.

All of the results of the long-term electret detectors, except those for the shortest exposure time of 1 d, produced small uncertainties (again expressed as standard deviation among replicate measurements). Voltage loss is 5 to 8 V when the long-term electret detectors are exposed for 1 d at a radon concentration of 41 pCi/L. Since the reader variability is expected to be ±2 V [4], the observed standard deviation at this low exposure level is consistent with that predicted by the manufacturer. At a greater concentration of radon, or a longer exposure time, reader variations become small relative to voltage changes, so uncertainties become correspondingly small. There was insufficient time prior to the meeting to test the long-term electret to its maximum integrated-exposure level.

## 3. Discussion

Our earlier conclusion [1] that charcoal detectors are unreliable for anything but the crudest of radon measurements appears to remain valid. Charcoal detectors cannot be recommended even for screening measurements, because any particular measurement may be nonrepresentative. Especially critical would be a situation where the measured value turns out to be lower than the actual concentration by such a large amount that the homeowner fails to take corrective action even though mitigation is warranted (see discussion in [1]).

It appears that Laboratory C has markedly improved its performance with ATDs from that reported previously [1]. However, the overall negative bias, the fact that 4 of 18 measurements were biased low by 25% or more, and the overall range in values found in our most recent study raise questions about using these detectors for recommendations of remedial action. The generally poor performance of ATD vendors in EPA’s proficiency testing program [5], coupled with Laboratory C’s performance in our studies, indicate that ATD vendors should be tested more comprehensively using double-blind proficiency samples.

The electret detectors impressed us with their ease of handling and rapidity of the measurement process as well as their generally excellent precision. The fact that we obtained meaningful results even when we tested the long- and short-term detectors at their limits for low exposure and high exposures, respectively, suggests that the electret detectors can provide reliable measurements when used properly. Especially important is that in most cases when errors occur, such as that resulting from a high rate of discharge, the result is a false positive and would likely be corrected when follow-up measurements are performed.

One important source of error with the electret detectors can occur if the electrets are used outside their respective exposure ranges. False-negative values could develop if care is not taken at very high radon concentrations or over excessively long exposure times. Since the electret detectors (as also the ATDs) actually measure the time-integrated exposure (pCi d/L), radon concentration is derived from the ratio of integrated exposure and the time of exposure. Unlike an ATD, which continues recording alpha tracks regardless of how long it is in place, an electret detector left in place after it is fully discharged could produce a false negative value.

Another important potential error in using the electret detectors would be to use them in a home for very short-term measurements, say of the order of a few days. Any short-term measurement with the electret detectors would suffer from short-term fluctuations in radon concentration, much as described for charcoal detectors [1]. The large day-to-day fluctuations of radon concentration in a home require reasonably long-term, integrated measurements regardless of which type of detector is used.

This discussion concerning electret detectors highlights in another way the fallacy of relying on charcoal detectors which begin losing some of their adsorbed radon immediately. Some, like the bag and the old Laboratory B detectors, fail after only 1 d of exposure; others after 2 or 3 d. At least with the electret detectors, a false negative would occur only after an excessive exposure, and the potential error would be easily recognized by the low charge reading at the end of exposure. With charcoal detectors there is no easy way to identify a false negative result.

Some other incidental concerns for the electret detectors, such as discharge during storage, were raised during the discussion following this presentation. However, these issues involve cost management and are not necessarily critical to risk management. An excessive discharge rate during measurement because of a high gamma-radiation field could produce a false-positive result if not properly corrected, but this is preferable to a false negative and can be corrected by simply measuring the gamma-dose rate when the detector is installed in a home [4].

The sources of error, especially for charcoal detectors and ATDs, apparently are not being corrected by the U.S. EPA proficiency-testing program, the deficiencies of which have been noted previously [1]. Separate “double-blind” studies performed by *The Patriot News*, a Harrisburg, Pennsylvania newspaper, and by Public Citizen—Buyers Up, a Washington, DC consumer-interest group, found that many companies certified by EPA reported results which were inconsistent and contradictory. Some laboratories failed even to duplicate their own results. Unfortunately, this supplemental information is in the popular press rather than the scientific literature. It does support our contention, however, that a much more comprehensive proficiency testing program is necessary in the United States.

Part of the reason for this symposium, especially the workshops, has been to explore various means for transferring the technology of radon measurements to any country desiring the technology. The four IPPP laboratories have successfully exhibited that standards can be made traceable across continents as well as across borders. However, the U.S. situation indicates that traceability alone may not be sufficient to assure the quality of radon measurements. The problem should not be dismissed as a peculiarity of an entrepreneurial economy. Whenever and wherever cost is a major consideration, measurement quality is likely to suffer. A comprehensive international quality assurance program needs to be developed.

There appears to be an important role to be served by the ICRM. It can provide a system to assure the quality of radon measurements by specifying which systems or detectors are acceptable. Also, it can develop or approve protocols for proficiency testing of measurement laboratories and/or systems. Most importantly, the ICRM can provide a framework for organizing intercomparisons wherein the tested laboratories are not aware of the significance of the submitted detector (i.e., by setting up a system for performing double-blind proficiency tests).

## Figures and Tables

**Table 1:** Radon concentrations obtained using Laboratory C alpha-track detectors. Uncertainty values have been calculated from Laboratory C’s reported percentage uncertainties, but the significant figures are consistent with those reported by Laboratory C. Reference values are listed as received from EML.

Exposure Time	Radon concentration (pCi/L±1 SD) by exposure time					
	1d	2d	3d	4d	5d	7d
	40.4±7,6	27.0±4.5	41.1±4.7	35.9±3.8	35.8±3.4	39.8±3.1
	37.0±7.3	35.8±5.2	35.4±4.3	38.8±4.0	29.7±3.1	32.8±2.9
	38.7±7.4	38.8±5.5	31.5±4.1	39.2±4.0	3S.5±3.5	35.4±2.9
Mean	38.7±1.7	33.9±6.1	36.0±4.8	38.0±1.8	34.5±4.5	36.0±3.5
Reference	41.1	41.8	42.5	42.0	44.4	43.6

**Table 2:** Radon concentrations measured using short- and long-term electret detectors. Reference values are reported as received from EML

Exposure time (days)	Radon concentration (pCi/L ±1 SD)^a^		
	Reference value	Short-term detectors	Long-term detectors
1	41.1	35.6±2.0	30.3±7.5
3	42.5	39.7±3.8	42.8 ±1.2^b^
4	42.0	40.3±0.9	41.0±2.3
5	44.4	43.6±3.3	42.1±2.1
7	43.6	42.6±0.9	41.9±0.6

a Mean of 3 measurements except as noted.

b Mean of 2 measurements.

## Footnotes

1 Certain commercial equipment, instruments, or materials are identified in this paper to specify adequately the experimental procedure. Such identification does not imply recommendation or endorsement by the National Institute of Standards and Technology, nor does it imply that the materials or equipment identified are necessarily the best available for the purpose. The views expressed in this article are solely those of the author and do not necessarily represent the views of the National Institute of Standards and Technology or the Department of Commerce. Neither NIST nor the DoC endorses or in any way approves the products or services of any company.
